# Protective Effects of Inhibition of Mitochondrial Fission on Organ Function After Sepsis

**DOI:** 10.3389/fphar.2021.712489

**Published:** 2021-09-08

**Authors:** Yu Zhu, Lei Kuang, Yue Wu, Haoyue Deng, Han She, Yuanqun Zhou, Jie Zhang, Liangming Liu, Tao Li

**Affiliations:** State Key Laboratory of Trauma, Burns and Combined Injury, Shock and Transfusion Department, Research Institute of Surgery, Daping Hospital, Army Medical University, Chongqing, China

**Keywords:** mitochondrial fission, Mdivi-1, Drp1, sepsis, organ function

## Abstract

Sepsis-associated organ dysfunction plays a critical role in its high mortality, mainly in connection with mitochondrial dysfunction. Whether the inhibition of mitochondrial fission is beneficial to sepsis-related organ dysfunction and underlying mechanisms are unknown. Cecal ligation and puncture induced sepsis in rats and dynamic related protein 1 knockout mice, lipopolysaccharide-treated vascular smooth muscle cells and cardiomyocytes, were used to explore the effects of inhibition of mitochondrial fission and specific mechanisms. Our study showed that mitochondrial fission inhibitor Mdivi-1 could antagonize sepsis-induced organ dysfunction including heart, vascular smooth muscle, liver, kidney, and intestinal functions, and prolonged animal survival. The further study showed that mitochondrial functions such as mitochondrial membrane potential, adenosine-triphosphate contents, reactive oxygen species, superoxide dismutase and malonaldehyde were recovered after Mdivi-1 administration via improving mitochondrial morphology. And sepsis-induced inflammation and apoptosis in heart and vascular smooth muscle were alleviated through inhibition of mitochondrial fission and mitochondrial function improvement. The parameter trends in lipopolysaccharide-stimulated cardiomyocytes and vascular smooth muscle cells were similar *in vivo*. Dynamic related protein 1 knockout preserved sepsis-induced organ dysfunction, and the animal survival was prolonged. Taken together, this finding provides a novel effective candidate therapy for severe sepsis/septic shock and other critical clinical diseases.

## Introduction

Sepsis is a systemic inflammatory response syndrome caused by a dysregulated host response to infection, leading to septic shock and even death. Substantial improvements have been achieved in the treatment of sepsis over the past decades, however, it is still associated with unacceptably high mortality (Lagu et al., 2012; Angus and van der Poll, 2013). Previous studies demonstrated that severe sepsis-induced multiple organ function disorder including liver, kidney and cardiovascular, play critical roles in the development of severe sepsis and septic shock accompanied by the increase of morbidity and mortality (Heemskerk et al., 2009; Schuler et al., 2018; Pang et al., 2019). At present, clinical treatments mainly focused on eliminating the causes, controlling infection and symptomatic measures, without specific intervention (Rhodes et al., 2017). It’s of crucial importance to explore a novel therapeutic approach for organ dysfunctions in sepsis.

Mitochondria are the important organelle, which maintains a dynamic process of continuous division and fusion. This dynamic process is vital for sustaining the normal structure and function of mitochondria, while the imbalance of mitochondrial fission and fusion is crucial for inducing mitochondrial dysfunction (Balog et al., 2016). Previous researches revealed that mitochondrial fission and fusion were regulated by mitochondrial fission proteins dynamic related protein 1 (Drp1) and fission1(Fis1), and mitochondrial fusion proteins mitofusin 1 (Mfn1), Mfn2, and optic atrophy1 (OPA1) (Watanabe et al., 2014). A previous study reported that the overexpression of mitochondrial fission protein Drp1 led to mitochondrial fragmentation and depolarization of the transmembrane potential in cardiomyocytes, and resulted in mitochondrial dysfunction (Lee and Yoon, 2016). Our previous studies found that mitochondria showed over-fission in cardiac muscle and vascular smooth muscle which induced mitochondrial dysfunction following hemorrhagic shock. (Duan et al., 2020). However, whether the inhibition of mitochondrial fission is beneficial to organ dysfunction in sepsis is unclear yet.

Basic research demonstrated that activation of mitochondrial fission protein Drp1 played a key role in the occurrence of mitochondrial fission. The Drp1 activity was modified by phosphorylation of different sites which resulted in its GTPase activation and then translocation to mitochondria (Taguchi et al., 2007). Mdivi-1 is a selective Drp1 inhibitor that could bind to the surface of the GTPase domain and disturb oligomeric assembly, and then inhibit the activation of Drp1 GTPase (Yang et al., 2020). Whether inhibition of mitochondrial fission with Mdivi-1 is beneficial to sepsis is not clear.

In the present study, cecal ligation and puncture (CLP)-induced sepsis in SD rats and Drp1 knockout Drp1 KO mice and lipopolysaccharide (LPS)-treated vascular smooth muscle cells (VSMCs) and cardiomyocytes were applied, the protective effects of inhibition of mitochondrial fission on organ function following sepsis and the underlying mechanisms were explored.

## Materials and Methods

### Drugs and Reagents

Mdivi-1 was obtained from Tocris bioscience (American). Lipopolysaccharide (LPS) were purchased from Sigma (St. Louis, MO, United States). H9C2 cell line was purchased from Biowit Technologies (Shenzhen, China). DMEM were from Gibco/Invitrogen. ATP content and JC-1 test kit were from Beyotime. Mito-Tracker™ Deep Red FM was obtained from Invitrogen. The kits of measurement of TNFα, IL-6, D-lactate, ROS, MDA, and SOD were from Nanjing Jiancheng Co., Ltd. ROS Detection Kit of cells were purchased from Abcam (Cambridge, MA, United States). Albumin-fluorescein isothiocyanate conjugate (FITC-BSA) and collagenase type Ⅱ were purchased from Sigma (St. Louis, MO, United States). NF-κ B p65 antibody (Abcam, 1:1000), NF-κ B p65 phosphorylation antibody (Abcam, 1:1000), GAPDH antibody (Thermo, 1:1000), β-actin antibody (Thermo, 1:7000). Tunel was purchased from Roche.

### Animal Ethical Approval

All animal care and experimental protocols were approved by the Research Council and Animal Care and Use Committee of Research Institute of Surgery, Daping Hospital, the Third Military Medical University (No. DHEC-2012-069), and according to the National Institutes of Health Guide for the Care and Use of Laboratory Animals. Efforts were made to minimize any pain or discomfort, and the minimum number of animals was used.

### Animals Preparation

Adult male and female Sprague-Dawley (SD) rats (200 **±** 10 g) were obtained from the Animal Centre, Daping Hospital, the Third Military Medical University. The rats were fed in a cage with a humidity of 40% and a temperature of 24 ± 1°C and light cycled (6 a.m.- 6 p.m.). Drp1 KO mice were generated by Shanghai Model Organisms Center, Inc. (Shanghai, CHINA) as described in our research team (Duan et al., 2020).

### Establishment of Sepsis Model

SD rats were anesthetized with sodium pentobarbital (30 mg/kg) preliminary and then added until the rats had no response to needle stimulus. The sepsis model was replicated by cecal ligation and puncture (CLP) as described previously (Zhu et al., 2016). Briefly, a laparotomy was performed, and the cecum was ligated and perforated with 0 silk needle from the position of 0.7 cm at the distal of the cecum. After the closure of the abdomen, the rats were placed back in the cages, after completing the CLP procedures, rats were allowed water and food *ad libitum*. After CLP 12 h, a PE catheter was inserted into the right femoral arteries for monitoring the mean arterial blood pressure (MAP) and the right femoral veins were catheterized for administration, the right carotid artery received a catheter for monitoring the cardiac function. To prevent clotting formation, the artery tubing was filled with normal saline with 500 U/ml of heparin. When the MAP was decreased to 70 mmHg or decreased by 30%, the sepsis model was established for subsequent experiments. The same procedure was also carried out on Drp1 KO mice.

### Cell Culture

The cell lines H9C2 were maintained in DMEM (Gibco) plus 10% FBS. VSMCs were obtained from the mesenteric arteries of SD rats by enzymatic digestion as described in our research team (Li et al., 2011). Before each experiment, VSMCs (3-5 passages) were serum-starved for 24 h.

### Isolation of Primary Adult Rat Cardiomyocytes

Primary adult cardiomyocytes were isolated from rats following an enzymatic digestion protocol. The hearts of adult rats were excised and perfused on a Langendorff apparatus with Tyrode solution without Ca^2+^ contained (mmol/L: NaCl 140.0, KCl 5.0, MgCl_2_ 1.0, HEPES 5.0, Glucose 10.0, pH 7.4), then digested with collagenase type II (240 U/mg) at 37°C for 20 min, the digested cardiomyocytes were stored in KB solution (KOH 120.0, L-Glutamic acid 120.0, KCl 10.0, KH_2_PO_4_ 10.0, MgSO_4_.7H_2_O 1.8, EGTA 0.5, Glucose 20.0, Taurine 10.0, HEPES 10.0, BSA 0.2%, pH 7.2). Before the experiment, the cardiomyocytes were gradually re-calcified to the physiological concentration.

### Measurements of Blood Flow, Blood Gases, Cardiac Output, Oxygen Delivery

SD rats underwent catheterization of the left ventricle and right external jugular vein for measurement of cardiac output (CO). CO was measured by a Cardiomax-III machine (Columbus Instruments, Columbus, Ohio). Cardiac index (CI) = CO ÷ Body surface area (S). Stroke index (SI) = CI÷HR. The values of oxygen delivery (DO_2_) and oxygen utilization (VO_2_) in tissue were calculated using the following equations: DO_2_ = CI×13.4×hemoglobin×arterial oxygen saturation (SaO_2_); VO_2_ = CI×13.4×Hemoglobin× [SaO_2_ -venous oxygen saturation (SvO_2_)]. Blood gases were measured by a blood gas analyzer (Phox plus L; Nova Biomedical, Waltham, Mass). The volume of the blood samples for the blood gases was 0.5 ml. To avoid additional blood loss for rats, an equal volume of blood was supplied after each sample was taken. The blood flows of liver, kidney, and intestines were measured by a Doppler imaging instrument (Peri Cam PSI ZR, Sweden), the liver and kidney were exposed to a laser at a distance of 14 cm. Color images were used to represent the relative perfusion level in specific parts, and the blood flow of the liver and kidney was analyzed by software (PIM soft).

### Echocardiography

Transthoracic echocardiography was performed on anesthetized rats using a Vivid 9 high-frequency color doppler ultrasound system (GE Healthcare, Boston, Unite States). Left ventricular systolic functions were measured in M-mode echocardiograms from the LV parasternal long-axis view. LV functional parameters, including fractional shortening (FS), ejection fraction (EF), were calculated.

### Vascular Permeability Measurement

Fluorescence albumin transmissivity in the tissue supernatant was used to measure the vascular permeability of the lungs. Rats were injected with fluorescent albumin (9 mg/kg) by the jugular vein, after 30 min, the abdomen was opened, the main abdominal vein was ligated, the abdominal aorta was cut off, and at the same time, normal saline was transfusion into the jugular vein until the lungs were flushed white. The left upper lobe of the lung was taken, weighed, and recorded the weight. The lung tissue was cut up and the 4 ml PBS was added, homogenate and centrifugal at 8,000 g × 4°C×10 min, taking supernatant, measuring fluorescence intensity at wavelength 500 nm, 530 nm with the spectrophotometer, with fluorescence intensity/lung quality representing pulmonary vascular permeability.

### Vascular Reactivity Measurement

The superior mesenteric artery was made into a 2–3 mm vascular ring, which was hung in the isolated organ perfusion injected with K-H solution (mM: NaCl 119.00, KCl 4.70, NaHCO_3_ 20.00, KH_2_PO_4_ 1.18, MgSO_4_ 1.17, EDTA-Na_2_ 0.03, CaCl_2_ 2.50, Glucose 11.00). The mixture of 95% O_2_ and 5% CO_2_ was continuously bubbled into the vessel. The initial tension was 0.5 g and incubated at 37°C for 2 h. After the tension curve was stable, the reference contraction was induced by 124 mmol/L K^+^. Until the curve reached the highest point, changing the K-H solution to continue equilibrium, the contractile reactivity of the vascular ring to norepinephrine (NE, 1 × 10^−9^-1×10^−4^ mol/L) was measured. After three washes, the artery ring was equilibrated for 40 min and the 10^−6^ mol/L concentrations of NE was added into the chamber to pre-contract the artery ring, followed by acetylcholine (Ach, 1 × 10^−9^-1×10^−4^ mol/L), and the relaxation reactivity of the artery ring was determined. The maximal systolic and diastolic responses (E _max_) were recorded under different NE and Ach concentrations.

### Mitochondrial Morphology Observation

The cardiomyocytes and VSMCs were inoculated in a confocal culture dish and grew to 60–70% fusion, which could be used for experiments. The cells medium was completely removed and washed 3 times with sterile PBS. After removing PBS, add MitoTracker™ Deep Red (100 nM) to the cell and incubate in a 37°C and 5% CO_2_ incubator for 30 min, the cells were imaged by laser confocal microscope (Leica TCS SP5, Germany). The red fluorescence excitation wave is 633 nm and the visible range is 655–670 nm.

### Cell ROS Fluorescence Probe Detection

When the cells grow to 50% fusion, dilute DCFH-DA with a serum-free medium according to 1:1000, and the final concentration is 10 μM. The cells were incubated in a 37°C incubator for 30 min, washed with serum-free culture medium 3 times to fully remove the DCFH-DA that did not enter the cells. The ROS fluorescence intensity was observed by the laser confocal microscope (fixed voltage 800 V). The excitation wave of green fluorescence is 488 nm, and the visible range is 501–563 nm. Quantification for the ROS assay was performed using Image J to measure the fluorescence intensity.

### Mitochondrial Membrane Potential

Cells were inoculated in a 20 mm diameter confocal culture dish with a density of 1×10^5^ cells. After washing the cells with PBS three times, the cells were stained with a JC-1 dye working solution (200×) and incubated at 37°C for 20 min. Laser confocal microscopy was used for observation. The maximum excitation wavelength of the JC-1 monomer (green) is 488 nm and the visible range is 501–563 nm. The maximum excitation wavelength of JC-1 polymer (red) is 633 nm and the visible range is 655–670 nm. Quantification for the ∆Ψm assay was carried out to measure the fluorescence intensity ratio of JC-1 aggregate/JC-1 monomer.

### TUNEL Staining

H9C2 and VSMCs were incubated on a 20 mm culture dish and fixed with 4% polyformaldehyde for 25 min. After washing twice with PBS for 5 min, cells were placed in the ice bath to break the cell membrane with 0.2% Triton X-100 for 5 min. After washing twice with PBS for 5 min each time, TUNEL reaction mixture was prepared: the experimental group was mixed with 50 μl TdT+ 450 μl d UTP solution with fluorescein-labeled, while the negative control group was only added with 50 μl d UTP solution with fluorescein-labeled, and the positive control group was added with 100 μl DNaseⅠ first, and the reaction was at 15–25°C × 10 min, and the following steps were the same as the experimental group. 50 μl TUNEL reaction mixture (only 50 μl d UTP solution with fluorescein-labeled in the negative control group) was added to the sample in a dark wet box for 37°C × 1 h. After washing three times, DAPI was added to stain nuclei. The apoptosis cells were conducted using Image J to quantify (the excitation wavelength was 450–500 nm and the detection wavelength was 515–565 nm).

### Transmission Electronic Microscopy Imaging

Fresh heart tissues were prepared according to the protocol described previously (Duan, et al., 2020). The samples were observed with a transmission electron microscope (H-7500, Hitachi Company, Japan).

### Statistical Analyses

Data were the means ± standard deviations (SD). Statistical differences were analyzed by a repeated measure one-way or two-way ANOVA followed by the post hoc Tukey test (SPSS17.0; SPSS Incorporated, Chicago, IL). Survival time and survival rate were analyzed by the median and interquartile range and Kaplan - Meier survival analysis and log-rank test. A *p* value less than 0.05 was considered statistically significant (two-tailed).

## Results

### Protective Effects of Mdivi-1 on Cardiovascular Functions in Septic Rats

Cardiovascular functions determined perfusion of organs, and the dysfunction led to multiple organ function disorder (Facundo et al., 2017). Cardiac muscle and vascular smooth muscle were used to explore the effects of inhibition of mitochondrial fission with Midivi-1. Septic rats received conventional treatment or administration of Mdivi-1 in conventional treatment. According to the guidelines of sepsis treatment (Rhodes et al., 2017), In the conventional treatment group, Lactated Ringer’s solution (LR, 50% blood volume) with an antibiotic (cefuroxime sodium, 100 mg/kg) and vasoactive drug (dopamine, 10  μg/kg/min) was administrated after CLP 12 h in Septic rats, the rats in Mdivi-1 group received Mdivi-1 (1 mg/kg or 3 mg/kg) in combination with conventional treatment.

**FIGURE 1:**
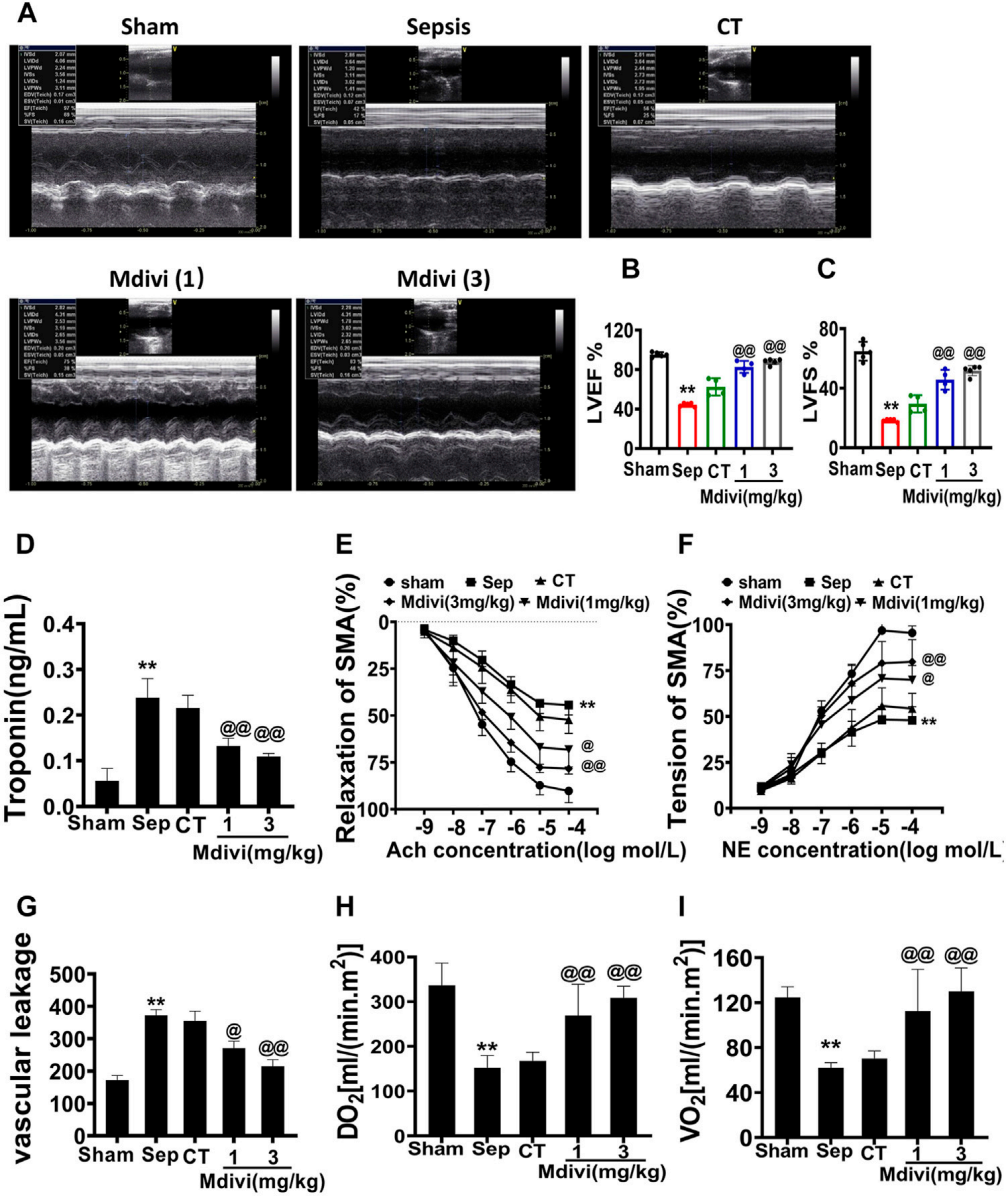
Effects of Mdivi-1 on cardiovascular function in septic rats. **(A–D)**: Effects of Mdivi-1 on cardiac function in septic rats. **(A)**: Echocardiograms. **(B,C)**: Summary of cardiac EF **(B)** and FS **(C)** measured using echocardiography. **(D)**: Effects of Mdivi-1 on blood troponin level in septic rats, the change of troponin, a marker of heart damage, was detected by blood sampling at the treatment point. **(E,F)**: Effects of Mdivi-1 on vascular contractile and relaxation function in septic rats. SMA was used to measure the contraction and relaxation responses to different concentrations of NE and Ach. **(G)**: Effects of Mdivi-1 on vascular leakage in septic rats. The pulmonary vein was taken and labeled with fluorescent albumin to measure the permeability of fluorescent albumin. **(H)**: DO_2_ (Oxygen delivery). **(I)**: VO_2_ (oxygen consumption). **p < 0.01 versus Sham. @*p* < 0.05 and @@*p* < 0.01 versus CT group. Sham = the control group, Sep = sepsis, CT = conventional treatment. 1: Mdivi-1 (1 mg/kg). 3: Mdivi-1 (3 mg/kg).

The cardiac function was severely impaired in septic rats, as evidenced by the decreases of CO, CI, SI (Supplementary Figures 1A–C) and the increase of cardiac muscle injury marker troponin. The conventional treatment could slightly improve CO, CI, SI and reduce troponin, but there was no significant difference as compared with sepsis. Mdivi-1 administration could significantly improve CO, CI, SI and reduce troponin, protective effects of 3 mg/kg of Mdivi-1 were better than 1 mg/kg. As compared with conventional treatment, CO, CI, and SI in the 3 mg/kg mdivi-1 group were increased by 64.7, 52.3, and 50.0% respectively (Supplementary Figures 1A–C). In addition, we used echocardiography to visualize the cardiac structures and evaluate cardiac function. The results showed that cardiac function was significantly impaired after sepsis, including both LVEF and LVFS. Both 1 mg/kg and 3 mg/kg Mdivi-1 significantly improved the systolic and diastolic functions of animals, and significantly increased LVEF and LVFS. Compared with conventional treatment, Mdivi-1 (3 mg/kg) increased by 40.8 and 75.6%, respectively **(** Figures 1A–D **)**.

Vascular dysfunction is an important factor affecting organ perfusion. Vascular hypo-reactivity could result in the decrease of tissue perfusion, vascular leakage led to numerous substances entering the tissue space which resulted in serious organ dysfunction. (Duan et al., 2015; Zhang et al., 2018). The effects of Mdivi-1 on vascular reactivity and vascular leakage were further studied in the present study. The results demonstrated that the vascular permeability of the pulmonary vein was increased significantly and the vascular reactivity was decreased after sepsis, which was shown that the permeability of the pulmonary vein to FITC was notably increased, and the contractile response of SMA to NE and the diastolic response to Ach were distinctly decreased. Conventional treatment slightly improved vascular hyper-permeability and vascular hypo-reactivity, but there was no significant difference as compared with sepsis. While 3 mg/kg Mdivi-1 had an evident protective effect on the vascular function, which could obviously reduce the vascular permeability and increase the vascular reactivity (Figures 1E–G).

Oxygen supply (DO_2_) and oxygen consumption (VO_2_) are the main factors influencing tissue and cell metabolism which is related to cardiovascular function. We further explored whether Mdivi-1 could improve the oxygen supply and consumption by improving cardiovascular function. The results showed that DO_2_ and VO_2_ in septic rats were significantly decreased, which decreased to less than 40% in the sham operation group. Following conventional treatment, DO_2_ and VO_2_ were partially increased. Compared with conventional treatment, DO_2_ and VO_2_ in the 3 mg/kg Mdivi-1 group respectively were increased by 89.4 and 90.3% (Figures 1H,I). The results indicate that Mdivi-1 protects sepsis-induced cardiovascular function disorder.

### Effects of Mdivi-1 on Vital Function and Survival in Septic Rats

**FIGURE 2:**
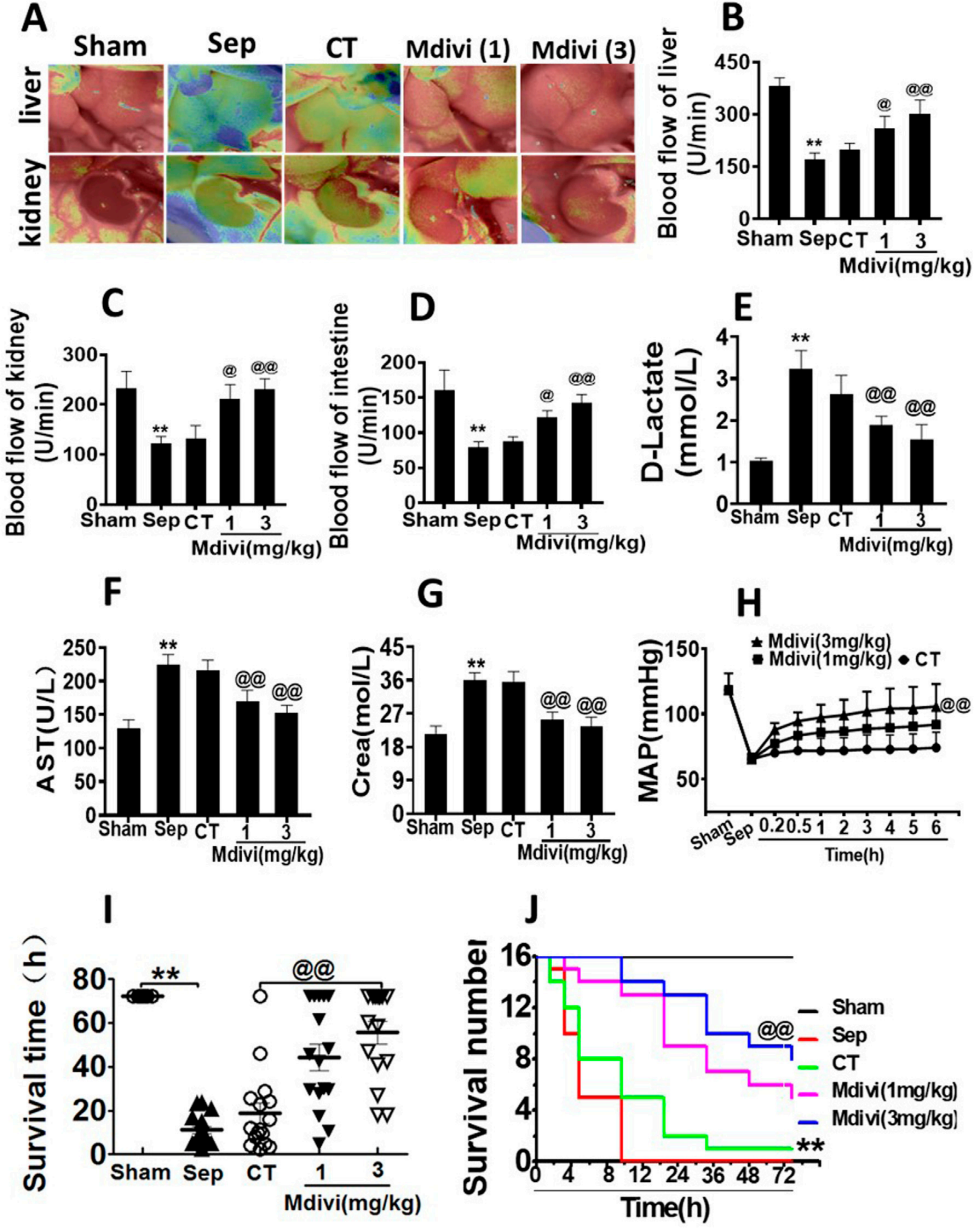
Effects of Mdivi-1 on vital organ function in septic rats. **(A)**: blood flow of liver and kidney with the Peri Cam PSI System. **(B–D)**: blood flow of liver, kidney and intestine with laser Doppler imaging. **(E)**: intestinal function (D-lactate). **(F)**: aspartate aminotransferase (AST). **(G)**: kidney function creatinine (Crea). **(H)**: Time-lapse monitoring of mean arterial pressure after Mdivi-1 treatment. After CLP 12 h, the mean arterial blood pressure (MAP) was monitored after administration of 12 min, 30 min, 1, 2, 3, 4, 5 and 6 h by femoral artery intubation. **(I, J)**: Effects of Mdivi-1 on survival in septic rats (n = 16). Rats were randomly divided into five groups, after 6 h of treatment, blood vessels were ligated, muscle and skin layers were sutured, and the average survival time and survival rate of rats within 72 h were observed. **p < 0.01 versus Sham. @p < 0.05 and @@p < 0.01 versus conventional treatment (CT) group. Sham, the control group; Sep, sepsis; CT, conventional treatment. 1: Mdivi-1 (1 mg/kg). 3: Mdivi-1 (3 mg/kg).

The above result showed that Mdivi-1improved cardiovascular function in septic rats, and the further study was focused on whether Mdivi-1 protected organ function by improving cardiovascular function. The blood flows of the liver, kidney, and intestine were measured by speckle and laser Doppler. It was shown that the perfusions of the liver, kidney, and intestine were decreased significantly after sepsis. Following conventional treatment, organ perfusions of the liver, kidney, and intestine were slightly recovered. While Mdivi-1 treatment obviously improved the blood flows of the organ. Compared with conventional treatment, the organ blood flows in the liver, kidney, and intestine were increased by 51.3, 73.0, and 56.0% respectively in the 3 mg/kg Mdivi-1 group (Figures 2A–D).

D-lactate is a metabolite of bacteria, and when acute ischemia or other injuries occur in the intestine, the blood plasma D-lactate level increases, thus monitoring the blood plasma D-lactate level reflects the degree of intestinal mucosal damage (Peoc’h et al., 2018) Aspartate aminotransferase (AST) and alanine aminotransferase (ALT) are mainly distributed in liver cells. Increased liver cell membrane permeability or necrosis of liver cells can enhance the levels of ALT and AST (Bertolini et al., 2020). Urea nitrogen (BUN) and creatinine (Crea) are the by-products of protein metabolism, which are mainly excreted by the kidneys. Crea and BUN are often used to assess the degree of renal damage (De Loor et al., 2013). In the following study, the effects of Mdivi-1 on the liver, kidney, and intestinal function were observed. The results showed that the levels of D-lactate, AST, ALT, BUN, and Crea were significantly increased after sepsis. Mdivi-1 treatment could alleviate the damage of the liver, kidney, and intestine functions. Compared with the conventional treatment, the treatment of 3 mg/kg Mdivi-1 had more distinct effect (Figures 2E–G; Table 1).

**TABLE 1:** Effects of Mdivi-1 on liver and kidney function in septic rats (*n* = 8).

Group	BUN (mmol/L)	ALT (U/L)
Sham	6.43 ± 0.99	36.23 ± 3.18
Sepsis	18.81 ± 2.54^**^	67.08 ± 4.59^**^
CT	16.07 ± 2.84	66.3 ± 2.29
Mdivi (1)	12.46 ± 1.81^@@^	52.35 ± 7.00^@^
Mdivi (3)	11.22 ± 1.63^@@^	48.96 ± 3.78^@@^

BUN: blood urea nitrogen, ALT: alanine transaminase. **p < 0.01 versus Sham. @*p* < 0.05 and @@*p* < 0.01 versus conventional treatment (CT) group. Sham = the control group, CT = conventional treatment. Mdivi (1): Mdivi-1 (1 mg/kg). Mdivi (3): Mdivi-1 (3 mg/kg).

Based on the above results, the effects of Mdivi-1 on animal survival were further observed in septic rats. The results showed that the average arterial pressure (MAP) was decreased to 65 mmHg after sepsis. The conventional treatment raised MAP to 74 mmHg, MAP after Mdivi-1 treatment could maintain a high level to 100 mmHg (Figure 2H). The survival results showed that more than half of the animals died within 12 h after sepsis, and none of the animals survived over 24 h, with an average survival time of about 8.83 h. After conventional treatment, the survival time was partially prolonged, while only one animal survived more than 72 h, and the average survival time was about 11.28 h. Mdivi-1 administration significantly prolonged the animal survival time, the average survival time was prolonged to 42.3 and 60.3 h respectively in 1 mg/kg and 3 mg/kg of Mdivi-1 group, and the 72 h survival rates were 5/16 and 8/16 (Figures 2I,J). The above results suggested that Mdivi-1 had a protective effect on sepsis by improving cardiovascular function.

### Effect of Mdivi-1 on Mitochondrial Morphology in Septic Rats

**FIGURE 3:**
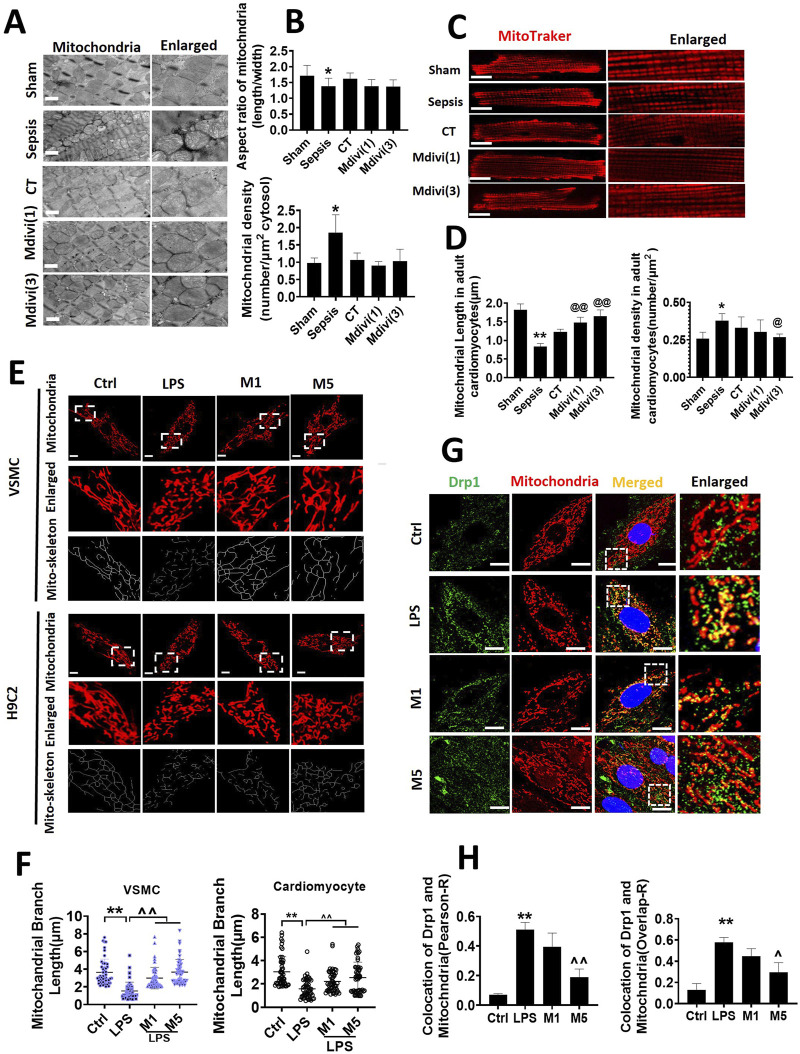
Effects of Mdivi-1 on mitochondrial morphology in septic rats. **(A, B)**: mitochondrial morphology of heart by transmission electron microscope (TEM) and statistical analysis (bar = 500 nm). **(C, D)**: mitochondrial morphology of acute isolation of cardiomyocytes *in vitro* and statistical analysis (bar = 25 μm). **(E, F)**: mitochondrial morphology of cardiomyocytes and vascular smooth muscle cell by laser confocal microscopy and statistical analysis (bar = 25 μm); **(G, H)**: colocalization of mitochondria and Drp1 (bar = 25 μm). *p < 0.05, **p < 0.01 versus sham or ctrl. @p < 0.05, @@p < 0.01 versus conventional treatment (CT) group. ^p < 0.05, ^p < 0.01 versus LPS. Sham; the control group; Sep, sepsis; CT, conventional treatment. 1: Mdivi-1 (1 mg/kg). 3: Mdivi-1 (3 mg/kg). H9C2: cardiomyocytes. VSMC: vascular smooth muscle cell. Ctrl: control group. M1: Mdivi-1 (10 μM). M5: Mdivi-1 (50 μM).

The effects on mitochondrial morphology were further observed in the present study. The observation of transmission electron microscope (TEM) showed that the mitochondria of cardiac muscle were orderly distributed along the sarcomere in the sham-operated group. The aspect ratio of mitochondria was about 1.71 ± 0.33, and the number of mitochondria was 0.97 ± 0.15/μm^2^. The arrangement of mitochondria in cardiac muscle was disordered and swelling after sepsis, the sarcomere and crista structure was disappeared, and the mitochondria were obviously smaller, the number of mitochondria was increased significantly, the aspect ratio was 1.38 ± 0.25 and the number was 1.86 ± 0.51/μm^2^. The mitochondrial morphology was partly improved following conventional treatment. After the administration of Mdivi-1, mitochondrial morphology and structure in cardiac muscle were notably restored with evenly arrangement and integral morphology, the number of mitochondria was decreased to 0.90 ± 0.12 and 1.03 ± 0.35 in 1 mg/kg and 3 mg/kg of the Mdivi-1 group (Figures 3A,B).

The acutely isolated cardiomyocytes were observed by a laser confocal microscopy after Mdivi-1 treatment in septic rats. The mitochondria in cardiomyocytes were orderly arranged, with long columnar mitochondria in the sham-operated group. Similar to the results of TEM, the mitochondria in cardiomyocytes from septic rats were in disorder with smaller volume, and the number of mitochondria was increased significantly. Conventional treatment did not obviously recover mitochondrial morphology. After the administration of Mdivi-1, the mitochondrial structure of cardiomyocytes was remarkably improved, with evenly arrangement and integral morphology, the number of mitochondria was decreased significantly. Among which the effect of Mdivi-1 (3 mg/kg) was much better (Figures 3C,D).

Furthermore, the morphology of mitochondria in LPS-stimulated cardiomyocytes and VSMCs was observed by confocal microscopy. The results showed that the structure of mitochondria in normal cardiomyocytes was long cord and evenly distributed, mitochondrial length was more than 3 µm. After LPS stimulation (1 μg/ml, 12 h), the mitochondrial structure was significantly fragmented, which were short and dotted distribution, mitochondrial length was reduced by 48.4%, dotted mitochondria were accounted for about 80% of all mitochondria. Both with 10 µM or 50 µM (for 4 h) of Mdivi-1 incubation alleviated the fragmentation of cardiomyocytes and restored the cords of mitochondria, mitochondrial length is increased by 40.8 and 62.4%. The changes of mitochondrial morphology in VSMCs after Mdivi-1 incubation were consistent with changes in cardiomyocytes (Figures 3E,F).

The distribution of Drp1 after Mdivi-1 treatment was further observed. Results showed that LPS increased Drp1 translocation from the cytoplasm to mitochondria both in cardiomyocytes and VSMCs, 50 μmol/L of Mdivi-1 treatment could antagonize LPS-induced the shift of Drp1 from cytoplasm to mitochondria (Figures 3G,H).

### Effect of Mdivi-1 on Mitochondrial Function in Septic Rats

**FIGURE 4:**
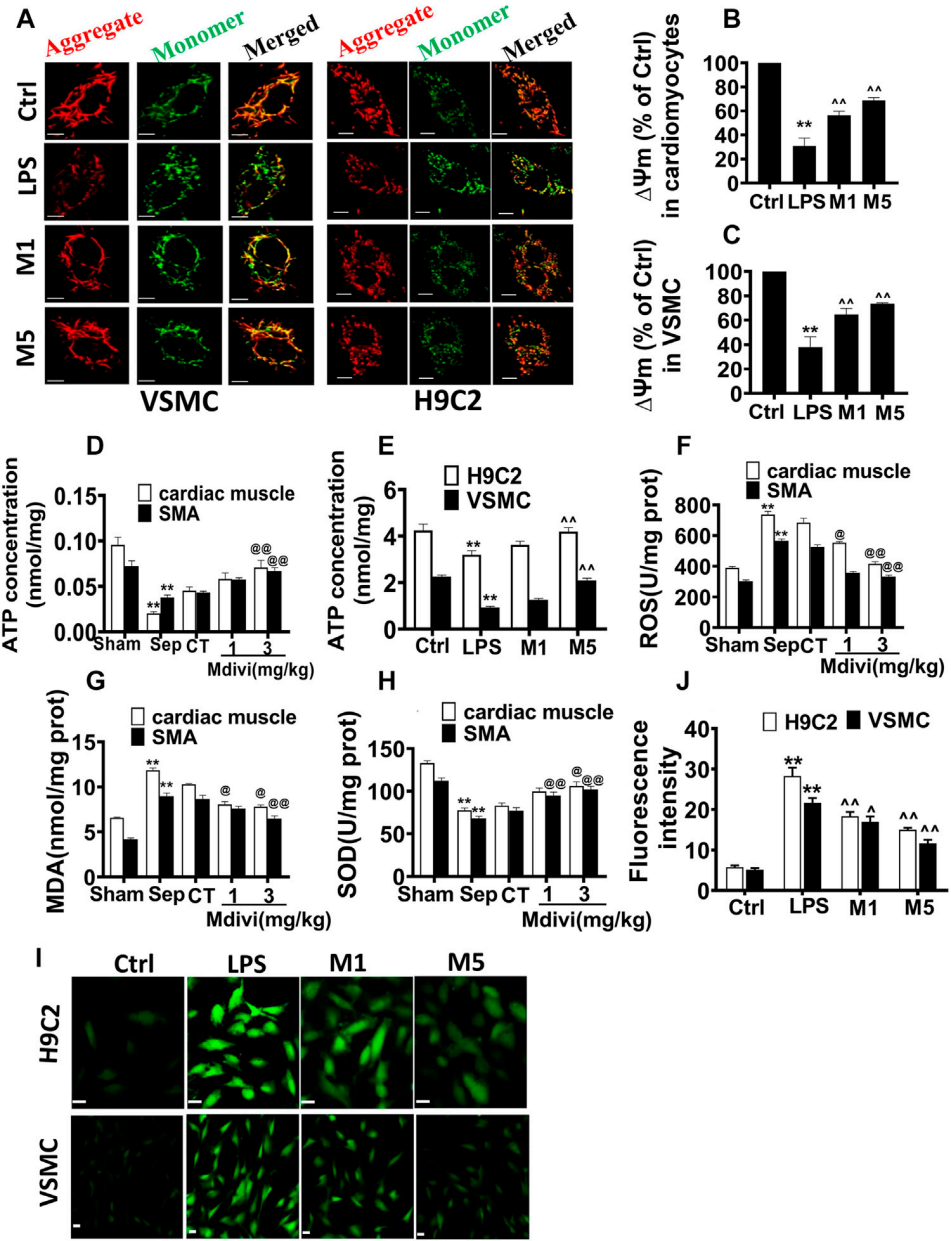
Effects of Mdivi-1 on mitochondrial function in septic rats. **(A–C)**: mitochondrial membrane potential of cardiomyocytes and vascular smooth muscle cells and statistical analysis (bar = 25 μm). **(D)**: ATP concentration of heart tissue and superior mesenteric arteria. **(E)**: ATP concentration of cardiomyocytes and vascular smooth muscle cells. **(F)**: ROS content in cardiac muscle and superior mesenteric arteria. **(G)**: MDA content in cardiac muscle and superior mesenteric arteria. **(H)**: SOD content in cardiac muscle and superior mesenteric arteria. **(I,J)**: ROS content in cardiomyocytes and vascular smooth muscle cell and statistical analysis (bar = 25 µm). **p < 0.01 versus sham or ctrl. @@*p* < 0.01 versus conventional treatment (CT) group. ^^ *p* < 0.01 versus LPS. Sham = the control group, Sep = sepsis, CT = conventional treatment. 1: Mdivi-1 (1 mg/kg). 3: Mdivi-1 (3 mg/kg). H9C2: cardiomyocytes. VSMC: vascular smooth muscle cell. Ctrl: control group. M1: Mdivi-1 (10 µM). M5: Mdivi-1 (50 µM).

The mitochondrial membrane potential (∆Ψm), ATP, and ROS were used to the representative of mitochondrial function. The results showed that LPS stimulation induced the decrease of ∆Ψm of cardiomyocyte and VSMC by 69.0 and 62.9%, respectively. Both 10 and 50 μM Mdivi-1 incubation recovered the ∆Ψm of cardiomyocyte and VSMC, and ∆Ψm was increased to 121.9 and 92.9% in cardiomyocytes and VSMCs after 50 µM Mdivi-1 treatment as compared with LPS stimulation (Figures 4A–C).

ATP contents in cardiac muscle and vascular smooth muscle artery were decreased significantly after sepsis. Conventional treatment could slightly elevate the ATP contents, Mdivi-1 administration exerted a more obvious role. Compared with the conventional treatment, 3 mg/kg Mdivi-1 increased the ATP contents by 57.0 and 55.8% in cardiac muscle and vascular smooth muscle artery respectively. The trends of ATP contents of cardiomyocytes and VSMCs following LPS and Mdivi-1 incubation were similar *in vivo* (Figures 4D,E).

The ROS was significantly increased in the heart and vascular tissue after sepsis, Mdivi-1 reduced the ROS level (Figure 4F). LPS (1 μg/ml) stimulation increased ROS production in cardiomyocytes and VSMCs, and the fluorescence intensities in cardiomyocytes and VSMCs were enhanced significantly. Both 10 and 50 μM Mdivi-1 incubation reduced the ROS level. Compared with the LPS group, 50 μM Mdivi-1 decreased the ROS production by 47.0 and 46.4% in cardiomyocytes and VSMCs individually **(** Figures 4I,J).

Malondialdehyde (MDA) was used as an important indicator to determinant the degree of lipid peroxidation and the level of free radicals in the body (Ayala et al., 2014). Superoxide dismutase (SOD) prevented the expansion of oxidative free radical chain reaction and was an important line of defense for the oxygen-free radical scavenging system in organisms (Bresciani et al., 2015). The effects of Mdivi-1 on the levels of MDA and SOD in heart and vascular smooth muscle tissue were further observed. The results showed that the levels of MDA in heart and vascular smooth muscle artery were increased significantly after sepsis. Mdivi-1 administration remarkably decreased MDA level compared to conventional treatment, 3 mg/kg Mdivi-1 decreased the MDA by 24.1 and 25.0% respectively in myocardial tissue and vascular smooth muscle tissue compared with conventional treatment **(** Figure 4G). Similarly, Mdivi-1 obviously antagonized sepsis-induced the decrease of SOD level, and 3 mg/kg Mdivi-1 increased by 27.7 and 32.6% in myocardial tissue and vascular smooth muscle tissue compared with conventional treatment **(** Figure 4H).

### Effects of Mdivi-1 on Inflammatory Response and Apoptosis in Cardiac Muscle and Vascular Smooth Muscle

**FIGURE 5:**
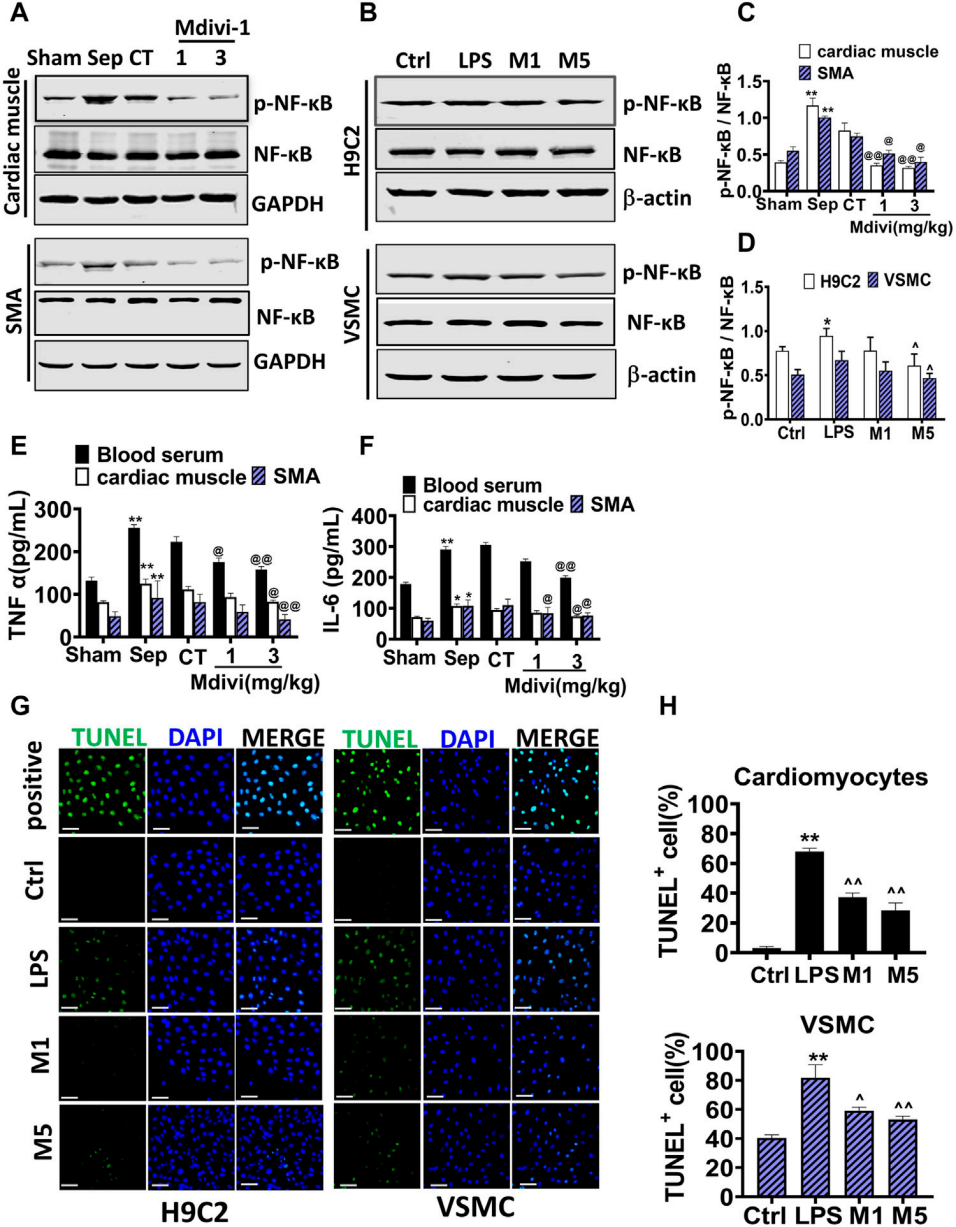
Effects of Mdivi-1 on inflammatory response and apoptosis in septic rats. **(A–D)**: Western blot and statistical analysis of the phosphorylation of NF-κ B. **(E)**: the concentration of TNF-α in blood serum, cardiac muscle and superior mesenteric arteria. **(F)**: the concentration of IL-6 in blood serum, cardiac muscle and superior mesenteric arteria. **(G,H)**: Effects of Mdivi-1 on apoptosis in cardiomyocytes and VSMC (bar = 50 µm). *p < 0.05, **p < 0.01 versus sham or ctrl. @*p* < 0.05 and @@*p* < 0.01 versus conventional treatment (CT) group. Sham = the control group, Sep = sepsis, CT = conventional treatment. 1: Mdivi-1 (1 mg/kg). 3: Mdivi-1 (3 mg/kg). SMA: superior mesenteric arteria. H9C2: cardiomyocytes. VSMC: vascular smooth muscle cell. Ctrl: control group. M1: Mdivi-1 (10 µM). M5: Mdivi-1 (50 µM).

The inflammation and apoptosis were major characteristics after sepsis and their occurrence was related to mitochondrial dysfunction (Deo et al., 2020). Therefore, inflammation and apoptosis in cardiac muscle and vascular smooth muscle were observed. The inflammatory factors including TNF-α, IL-6 were increased significantly after sepsis, Mdivi-1 distinctly reduced the levels of TNF-α, IL-6 in serum, myocardial tissue, and vascular smooth muscle tissue (Figures 5E,F), and the phosphorylation of NF-κ B p65 in myocardial tissue and vascular smooth muscle tissue (Figures 5A,C). In cardiomyocytes and VSMCs, Mdivi-1 incubation evidently reduced the LPS-induced phosphorylation of NF-κ B p65 (Figures 5B,D). LPS stimulation increased the apoptotic bodies of cardiomyocytes and VSMCs. Mdivi-1 incubation abolished the increase of apoptotic bodies induced by LPS stimulation (Figures 5G,H). The results indicated that Mdivi-1 had a significant protective effect on inflammation and apoptosis in the myocardium and vascular smooth muscle.

### Protective Effect of Drp1 Knockout on Septic Mice

The above results showed that the inhibitor of mitochondrial fission, Mdivi-1, had a significant protective effect on sepsis. To further elucidate the effects of inhibition of mitochondria on sepsis, Drp1 KO mice were used to further observed. Previous studies demonstrated that homozygous knockout mice could not survive, thus heterozygous knockout mice were used in this experiment. There were no obvious changes in body weight and basic vital signs including MAP, respiration, and heart rate in the Drp1 KO mice compared with wild-type (WT) mice.

**FIGURE 6:**
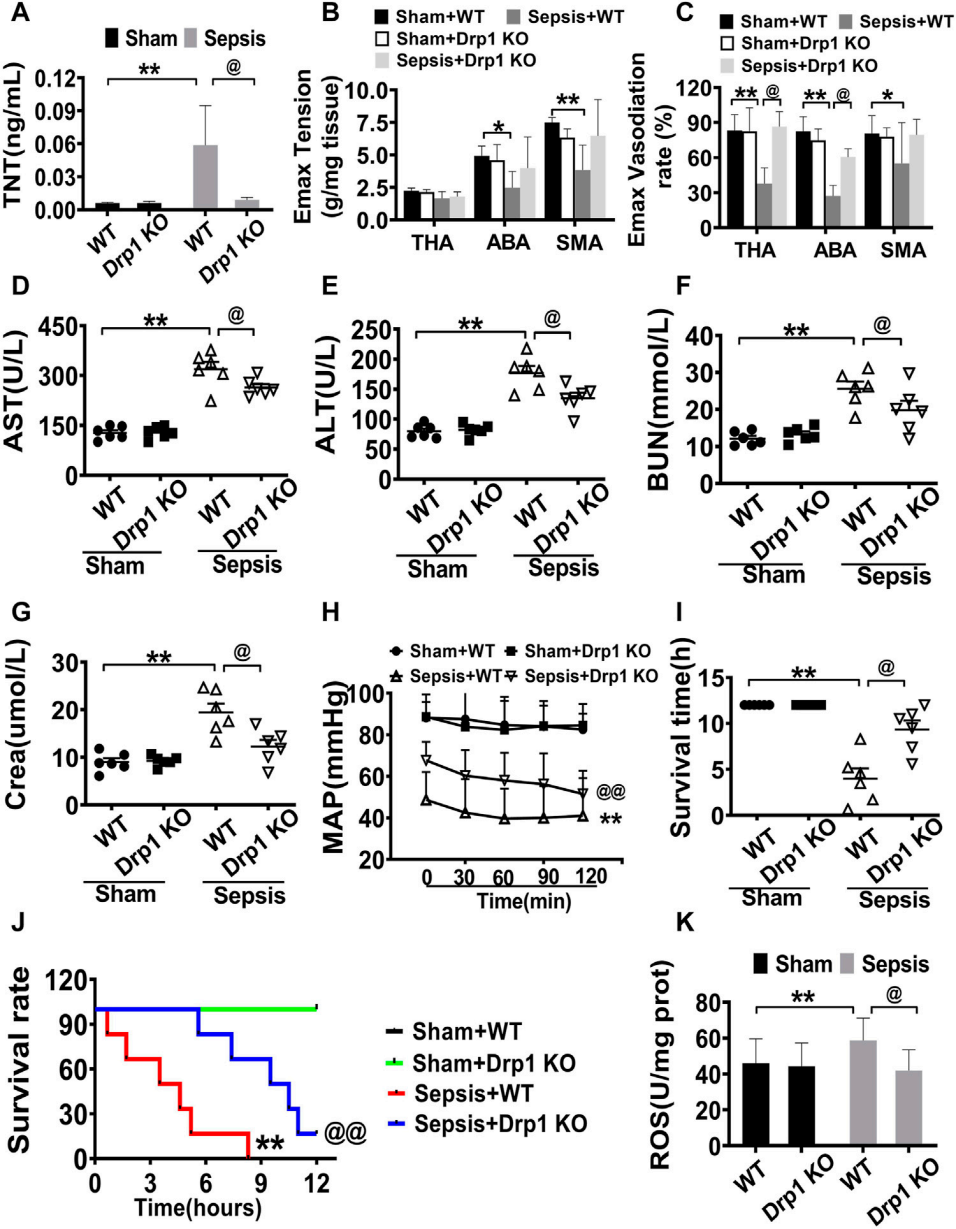
Protective effects of Drp1 knockout in septic mice. **(A)**: troponin T, a marker of heart damage. **(B,C)**: Effects of Mdivi-1 on vascular contractile and relaxation function in Drp1 KO mice. **(D)**: AST (aspartate aminotransferase). **(E)**: ALT (alanine transaminase). **(F)**: BUN (blood urea nitrogen). **(G)**: Crea (creatinine). **(H)**: MAP (mean arterial pressure). **(I)**: survival time. **(J)**: survival rate. **(K)**: ROS content in cardiac muscle. **p < 0.01 versus Sham of wild type. @*p* < 0.05, @@*p* < 0.01 versus sepsis of wild type. WT = wild type, Drp1 KO = Drp1 knockout. TNT = troponin T. ABA: abdominal aorta. THA: thoracic aorta. SMA: superior mesenteric arteria.

The level of troponin-T (TNT), the vascular contractile response, and vasodilation of the thoracic aorta, abdominal aorta, and the branches of the mesenteric artery in Drp1 KO mice after sepsis were measured. The results showed that the levels of TNT before sepsis were no different between Drp1 KO and WT mice, and the levels of TNT after sepsis were increased both in Drp1 KO mice and WT mice, while the increase ratio of TNT level in Drp1 KO was lower than WT after sepsis (Figure 6A). And similar to changes of TNT, the vascular contractile response and diastolic function were preserved in Drp1 KO suffering from sepsis. In WT mice, the contractile and diastolic response in the thoracic aorta, abdominal aorta, and the branches of the mesenteric artery of mice were decreased significantly after sepsis. While the damage of contractile or diastolic response in Drp1 KO mice was not alleviated compared with WT mice (Figures 6B,C). The results indicated that Drp1 KO had a protective effect on sepsis-induced cardiovascular function disorder.

The levels of AST, ALT, BUN, and Crea in Drp1 KO mice and WT mice were similar without sepsis. The levels of AST, ALT, BUN, and Crea were increased both in Drp1 KO mice and WT mice, while the increased ratio of these parameters in Drp1 KO mice was less than those in WT mice. Compared with WT mice, the levels of AST, ALT, BUN, and Crea were lower by 17.3, 23.7, 22.6 and 37.0%, respectively in Drp1 KO mice (Figures 6D–G). The results indicated that Drp1 KO also had a good protective effect on vital organ functions suffering from sepsis.

Based on the above results, the MAP and survival time was further observed in Drp1 KO mice. The results showed that without sepsis, the MAP was maintained at 90 mmHg both in the WT and Drp1 KO mice. After sepsis, MAP was decreased both in Drp1 KO and WT mice, while the MAP was maintained at 45 mmHg in WT mice, and the MAP in Drp1 KO was above 60 mmHg (Figure 6H). All WT mice died within 9.0 h after sepsis, and the average survival time was about 4.0 h. The survival time of Drp1 KO suffering from septic mice was 9.3 h and the 8 h survival rate was 66.7%. (Figures 6I,J). The results showed that Drp1 KO could prolong the survival time and improve the survival rate of sepsis mice.

The changes of ROS were observed in Drp1 KO mice. The results showed that ROS was significantly increased in WT mice suffering from sepsis, Drp1 KO inhibited sepsis-induced the increase of the level of ROS. ROS in Drp1 KO mice was lower than that in WT mice following sepsis, indicating that Drp1 KO could abolish sepsis-induced the increase of ROS (Figure 6K).

## Discussion

Mitochondrion is the main place of aerobic respiration in cells, and the changes of its structure and function play an important role in cell function. Previous studies demonstrated that mitochondrial dysfunction played a crucial part in multiple organ dysfunctions after critical illness, and the degree of mitochondrial dysfunction determined the outcome of patients (Annesley and Fisher, 2019). The present study demonstrated that inhibition of mitochondrial fission with Mdivi-1 had a protective effect on sepsis-induced organ function disorder and decreased the mortality of sepsis. The mechanisms were related to Mdivi-1 recovering mitochondrial morphology and improving mitochondrial function, and then alleviating oxidative damage, inflammation, and apoptosis. Our studies also found that Drp1 KO preserved sepsis-induced organ dysfunction. Present results provided a hopeful treatment for sepsis.

In view of the important role of mitochondrial function in organ function, some measures by improving mitochondrial function had been proposed in the past, such as Mito Q (Tauskela, 2007), SS31 (Ding et al., 2021), and resveratrol (Jardim et al., 2018), etc., but these measures had not achieved the desired effects. Due to the decisive role of mitochondrial morphology on mitochondrial function, the present study confirmed that inhibiting mitochondrial division significantly improved mitochondrial function, increased membrane potential, and ATP production, and significantly decreased ROS and MDA. Our study demonstrated that inhibiting excessive mitochondrial division provided an important direction to protect mitochondrial function and organ function after sepsis.

A large of studies showed that Mdivi-1 was a classic mitochondrial division inhibitor, which reduced excessive mitochondrial division by reducing the activity of GTPase Drp1 (Koch and Traven, 2019). The study demonstrated that Mdivi-1 could inhibit the translocation of Drp1 to mitochondria and inhibited mitochondrial division by inhibiting the activity of Drp1 (Sharp et al., 2014) (Manczak et al., 2019). Our previous study further found that Mdivi-1 inhibited mitochondrial division by inhibiting the phosphorylation of Drp1 at position 616 (Duan et al., 2020). However, some studies found that Mdivi-1 was not limited to inhibit mitochondrial division, but also participated in other functions. For example, Mdivi-1 could inhibit mitochondrial respiratory chain complex I and reduce the generation of ROS in the case of Drp1 deficiency (Duan et al., 2020). In addition, other inhibitors of mitochondrial fission had been developed, such as P110, which could improve mitochondrial function and reduce oxidative stress damage, improve mitochondrial membrane potential and inhibit the release of cytochrome c (Qi et al., 2013; Su and Qi, 2013). The detailed mechanisms of Mdivi-1 on sepsis should be further elucidated.

In the present study, we found that Drp1 KO mice had an obvious effect on resisting sepsis. The survival time and survival rate were prolonged, and organ function was protected in Drp1 KO mice suffering from sepsis. The Drp1 KO mice used in this experiment were partial knockout of Drp1, not full gene knockout. Because the full gene knockout mice could not survive and the experiments were unable to be followed. It had been reported that the knockout of Drp1 homozygote led to abnormal development and even death of the embryo. Partial knockout of Drp1 did not show any obvious physiological or behavioral abnormalities, as well as the lethality of the embryo [(Qi et al., 2019)]. In the present experiment, the knockout efficiency of Drp1 was about 50%, the basic vital signs of knockout mice had no significant change, and the blood pressure, respiration, and heart rate were normal.

Mitochondrial fission was mainly mediated by Drp1 activation. After activation, Drp1 trans-located from the cytoplasm to mitochondrion, and binds with receptors FIS1, MFF, and MID49/51 on the mitochondrial membrane, triggering mitochondrial fission (Rovira-Llopis et al., 2017). Under physiological conditions, Drp1 was mainly distributed in the cytoplasm, once stimulated, it transferred from the cytoplasm to the mitochondria (Ji et al., 2017). We found that LPS increased the aggregation of Drp1 on mitochondria, and these effects were inhibited by Mdivi-1, co-localizations between mitochondria and Drp1 were decreased. Previous studies demonstrated that the activity of Drp1 was modulated by its phosphorylation. Phosphorylation of serine at position 637 of Drp1 promoted Drp1 activation and mitochondrial fission, the phosphorylation of serine at position 637 or 656 of Drp1 inactivated Drp1 and inhibited mitochondrial fission (Flippo et al., 2020). The studies also found that Mdivi-1 inhibited the dephosphorylation of serine at the 637 position of Drp1 and protected the structure of myocardial mitochondria (Zaja et al., 2014). A variety of protein kinases, including CaMKII, Cyclins, and CDK, could phosphorylate Drp1, resulted in the activation of Drp1 and mitochondrial fission (Buhlman et al., 2014; Godoy et al., 2014; Jahani-Asl et al., 2015; Preau et al., 2016; Prieto et al., 2016). The other studies found that Drp1 ubiquitination and hematoxylin also induced Drp1 activation (Braschi et al., 2009). The specific changes of activation and phosphorylation sites of Drp1 after sepsis need to be further studied.

The present study found that inhibition of mitochondrial division alleviated sepsis-induced inflammation, including the release of cytokines TNFα and IL-6, and the phosphorylation of NF-κB. It had been proposed that mitochondrial dysfunction led to the activation of the nod-like receptor family pyrin domain-containing 3 (NLRP3) through mitochondrial ROS (mt ROS). Zhou reported that Mdivi-1 treatment decreased the expression of pro-inflammatory cytokines and activated (NLRP3) inflammasome and NF-κB (Zhou et al., 2020). Previous studies showed that dysfunctional mitochondria induced and enhanced inflammatory responses by producing mitochondrial ROS and releasing mitochondrial damage-associated molecular patterns (DAMPs) (van Horssen et al., 2019), whether Mdivi-1 is related to DAMPs in alleviating inflammation are needed to further investigation.

**FIGURE 7:**
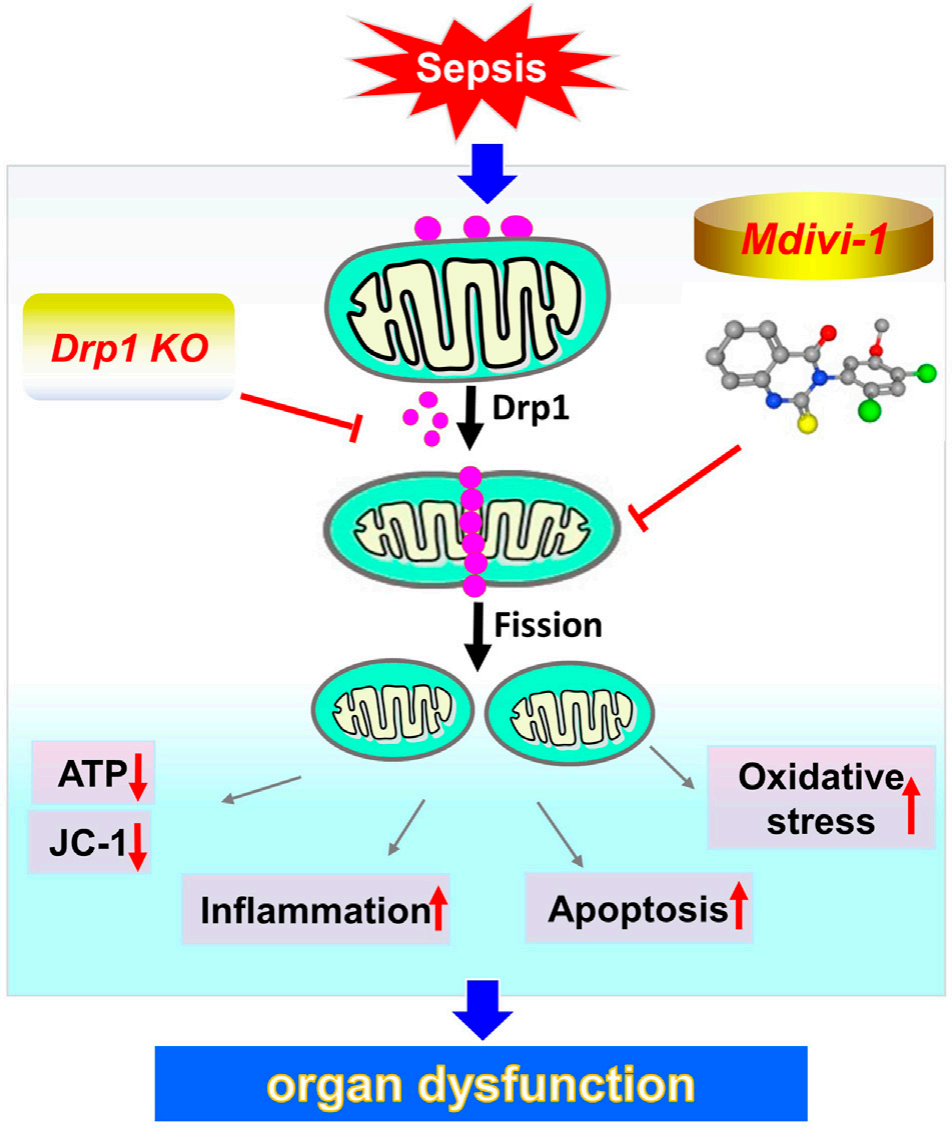
Schematic depiction of the potential protective effect of inhibition of mitochondrial fission during sepsis.

Mitochondria are the energy metabolism centers of cells and control the life and death of cells. Mitochondrial dysfunction is closely related to the occurrence of diseases (Johnson et al., 2021). Disorder of mitochondrial structure induced mitochondrial dysfunction and triggered cell apoptosis (Xiong et al., 2014). Previous studies showed that oxidative stress disturbed mitochondrial membrane potential, caused mitochondrial swelling and rupture of the outer membrane, released pro-apoptotic proteins, resulted in irreversible cell damage and death (Kroemer et al., 2007). Our study found that inhibiting mitochondrial fission reduced apoptosis. The possible mechanism was that Mdivi-1 inhibited the opening of MPTP channels and reduced the release of pro-apoptotic proteins. Our previous studies demonstrated that inhibition of Drp1 decreased the MPTP opening. Givvimani’s study also revealed that Mdivi-1 could inhibit the apoptosis of cardiomyocytes, the mechanism was related to reducing the level of ROS by inhibiting the division of mitochondria and reducing cell apoptosis (Givvimani et al., 2012). In a word, the present study demonstrated that inhibition of mitochondrial fission had a good protective effect on sepsis, which protected important organ functions by inhibiting oxidative stress, inflammatory response, and apoptosis (Figure 7). This finding provides a hopeful treatment for severe sepsis/septic shock and other critical clinical diseases.

In addition, the current study had some limitations. First, all experiments were performed in septic rats and mice, whether it is suitable for septic patients needs to be further studied. Second, our study found that inhibiting mitochondrial division inhibited Drp1 translocation to mitochondria, but the specific mechanisms of Drp1 activation and Drp1 modification after sepsis need further investigation. Third, we will focus on how inhibition of mitochondrial division inhibits inflammation and apoptosis.

## Data Availability

The original contributions presented in the study are included in the article/Supplementary Material, further inquiries can be directed to the corresponding authors.
