# Corrigendum: Protective effects of inhibition of mitochondrial fission on organ function after sepsis

**DOI:** 10.3389/fphar.2024.1493530

**Published:** 2024-10-09

**Authors:** Yu Zhu, Lei Kuang, Yue Wu, Haoyue Deng, Han She, Yuanqun Zhou, Jie Zhang, Liangming Liu, Tao Li

**Affiliations:** State Key Laboratory of Trauma, Burns and Combined Injury, Shock and Transfusion Department, Research Institute of Surgery, Daping Hospital, Army Medical University, Chongqing, China

**Keywords:** mitochondrial fission, Mdivi-1, Drp1, sepsis, organ function

In the published article, there was an error in Figure 2 as published. Figure 2A depicts blood flow experiments. Upon comparison, we confirmed the images “Sep” and “Mdivi (1)” in the “kidney” blood flow to be identical images; specifically, the image of “Mdivi (1)” in the “kidney” blood flow was misused**.** The corrected Figure 2 and its caption appear below.

**FIGURE 2:**
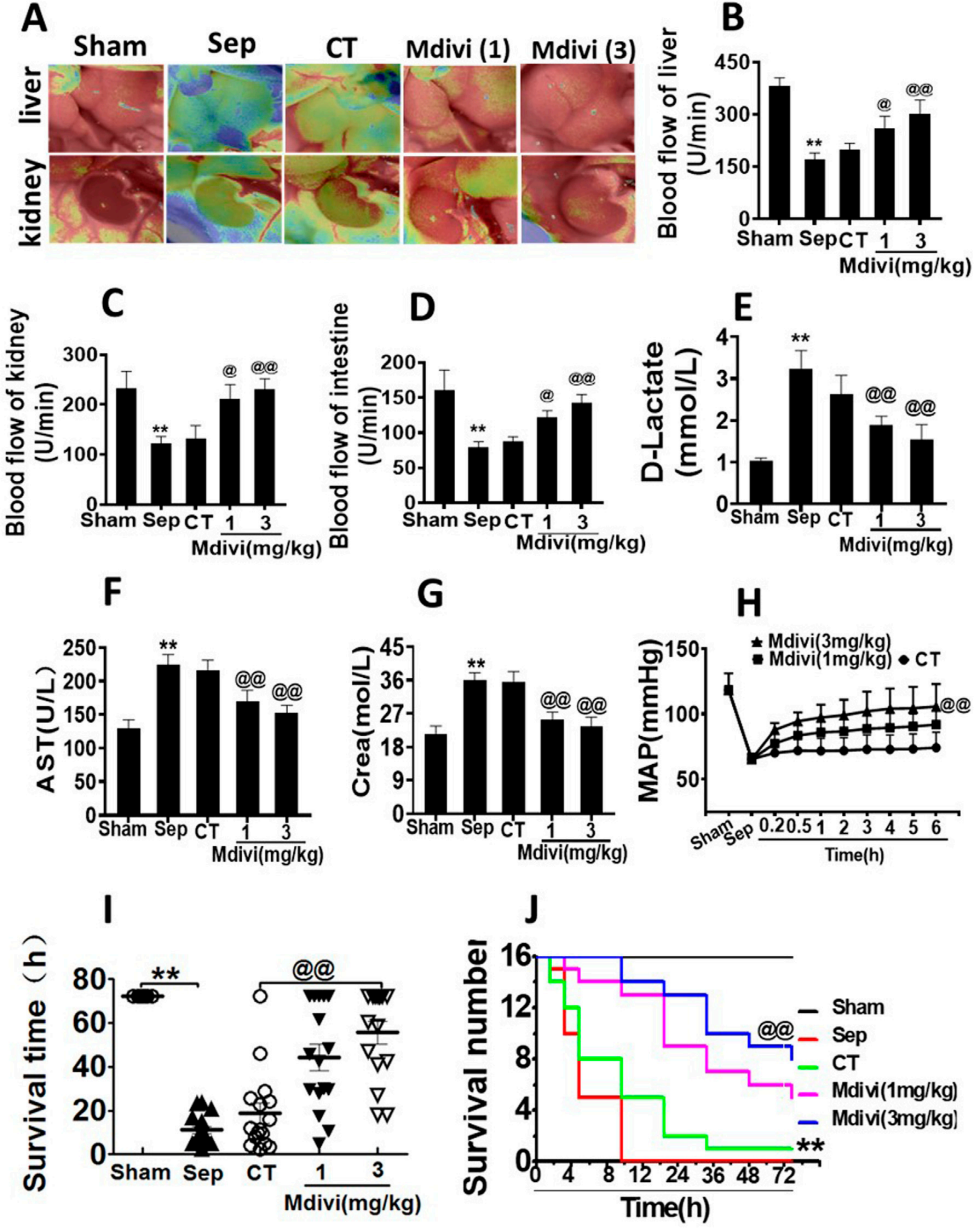
Effects of Mdivi-1 on vital organ function in septic rats. **(A)**: blood flow of liver and kidney with the Peri Cam PSI System. **(B–D)**: blood flow of liver, kidney and intestine with laser Doppler imaging. **(E)**: intestinal function (D-lactate). **(F)**: aspartate aminotransferase (AST). **(G)**: kidney function creatinine (Crea). **(H)**: Time-lapse monitoring of mean arterial pressure after Mdivi-1 treatment. After CLP 12 h, the mean arterial blood pressure (MAP) was monitored after administration of 12 min, 30 min, 1, 2, 3, 4, 5 and 6 h by femoral artery intubation. **(I, J)**: Effects of Mdivi-1 on survival in septic rats (n = 16). Rats were randomly divided into five groups, after 6 h of treatment, blood vessels were ligated, muscle and skin layers were sutured, and the average survival time and survival rate of rats within 72 h were observed. **p < 0.01 versus Sham. @p < 0.05 and @@p < 0.01 versus conventional treatment (CT) group. Sham, the control group; Sep, sepsis; CT, conventional treatment. 1: Mdivi-1 (1 mg/kg). 3: Mdivi-1 (3 mg/kg).

In the published article, there was an error in Figure 3 as published. Figure 3C depicts mitochondrial morphology experiments of acutely isolated cardiomyocytes. Upon comparison, we confirmed the images in “CT”and “Mdivi (1)” to be identical images; specifically, the image of “Mdivi (1)” was misused**.** The corrected Figure 3 and its caption appear below.

**FIGURE 3:**
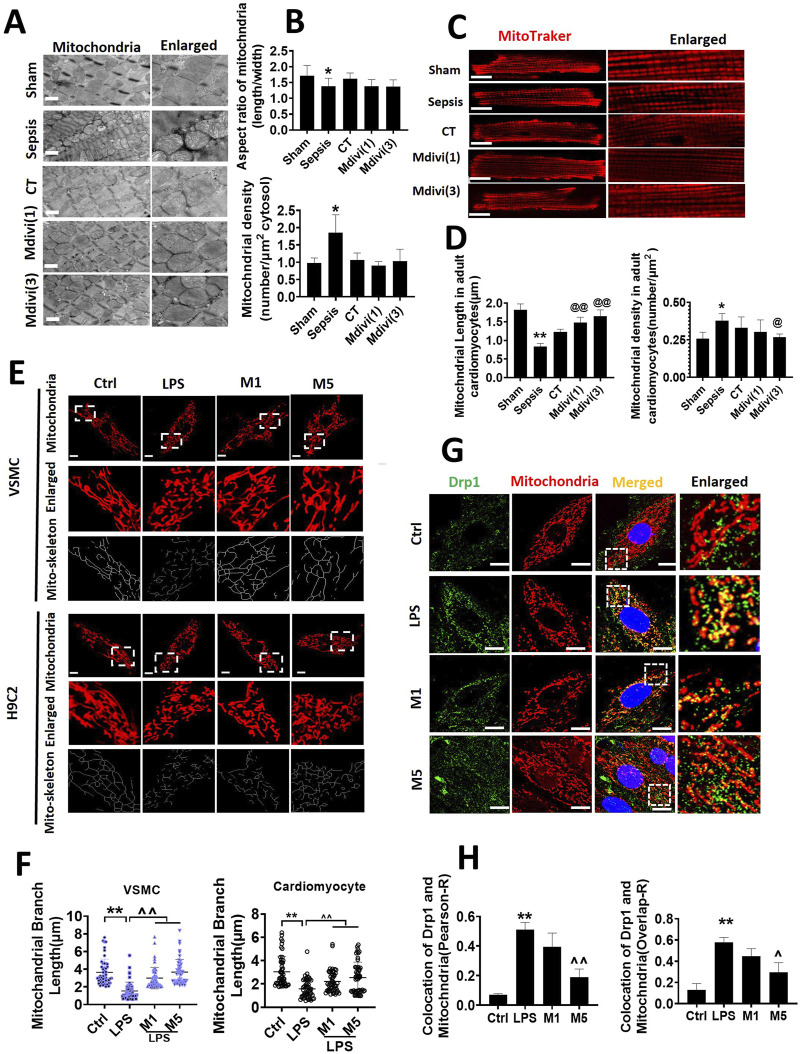
Effects of Mdivi-1 on mitochondrial morphology in septic rats. **(A, B)**: mitochondrial morphology of heart by transmission electron microscope (TEM) and statistical analysis (bar = 500 nm). **(C, D)**: mitochondrial morphology of acute isolation of cardiomyocytes *in vitro* and statistical analysis (bar = 25 μm). **(E, F)**: mitochondrial morphology of cardiomyocytes and vascular smooth muscle cell by laser confocal microscopy and statistical analysis (bar = 25 μm); **(G, H)**: colocalization of mitochondria and Drp1 (bar = 25 μm). *p < 0.05, **p < 0.01 versus sham or ctrl. @p < 0.05, @@p < 0.01 versus conventional treatment (CT) group.^p < 0.05,^p < 0.01 versus LPS. Sham; the control group; Sep, sepsis; CT, conventional treatment. 1: Mdivi-1 (1 mg/kg). 3: Mdivi-1 (3 mg/kg). H9C2: cardiomyocytes. VSMC: vascular smooth muscle cell. Ctrl: control group. M1: Mdivi-1 (10 μM). M5: Mdivi-1 (50 μM).

The authors apologize for these errors and state that this does not change the scientific conclusions of the article in any way. The original article has been updated.

