# Supplementary material for: Protective Effects of Inhibition of Mitochondrial Fission on Organ Function After Sepsis
Source: Front Pharmacol. 2021 Sep 8;12:712489. doi: 10.3389/fphar.2021.712489 (PMC8457550; doi:10.3389/fphar.2021.712489)
Supplement: Supplementary file 1 [file Image1.pdf]

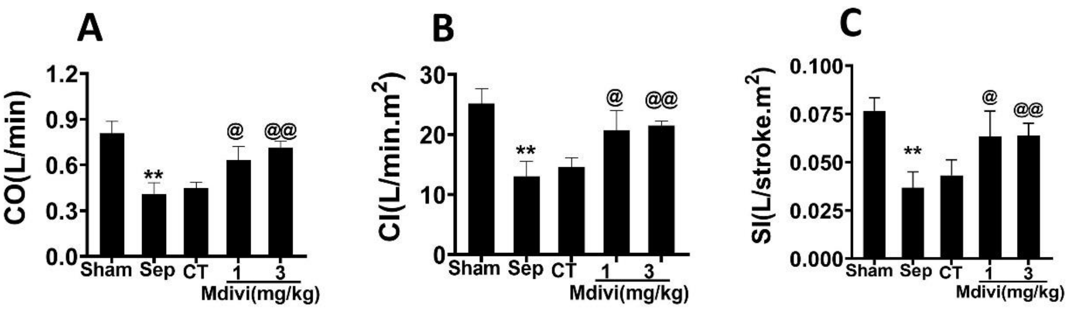

SUPPLEMENTAL FIGURE 1. Effects of Mdivi-1 on cardiac function in sepsis rats. **(A)**: CO **(**cardiac output), **(B)**: CI (cardiac index). **(C)**: SI (stroke index). **p < 0.01 versus Sham. @p < 0.05 and @@p < 0.01 versus CT group. Sham=the control group, Sep=sepsis, CT=conventional treatment. 1: Mdivi-1 (1mg/kg). 3: Mdivi-1 (3mg/kg).
